# Prognostic Impact of Multiple Lymphocyte-Based Inflammatory Indices in Acute Coronary Syndrome Patients

**DOI:** 10.3389/fcvm.2022.811790

**Published:** 2022-05-03

**Authors:** Qiuxuan Li, Xiaoteng Ma, Qiaoyu Shao, Zhiqiang Yang, Yufei Wang, Fei Gao, Yujie Zhou, Lixia Yang, Zhijian Wang

**Affiliations:** Beijing Key Laboratory of Precision Medicine of Coronary Atherosclerotic Disease, Department of Cardiology, Beijing Anzhen Hospital, Clinical Center for Coronary Heart Disease, Beijing Institute of Heart Lung and Blood Vessel Disease, Capital Medical University, Beijing, China

**Keywords:** acute coronary syndrome, percutaneous coronary intervention, lymphocyte-based inflammatory indices, GRACE risk score, major adverse cardiovascular events

## Abstract

**Background:**

The aim of this study was to evaluate the prognostic values of five lymphocyte-based inflammatory indices (platelet-lymphocyte ratio [PLR], neutrophil-lymphocyte ratio [NLR], monocyte-lymphocyte ratio [MLR], systemic immune inflammation index [SII], and system inflammation response index [SIRI]) in patients with acute coronary syndrome (ACS).

**Methods:**

A total of 1,701 ACS patients who underwent percutaneous coronary intervention (PCI) were included in this study and followed up for major adverse cardiovascular events (MACE) including all-cause death, non-fatal ischemic stroke, and non-fatal myocardial infarction. The five indices were stratified by the optimal cutoff value for comparison. The association between each of the lymphocyte-based inflammatory indices and MACE was assessed by the Cox proportional hazards regression analysis.

**Results:**

During the median follow-up of 30 months, 107 (6.3%) MACE were identified. The multivariate COX analysis showed that all five indices were independent predictors of MACE, and SIRI seemingly performed best (Hazard ratio [HR]: 3.847; 95% confidence interval [CI]: [2.623–5.641]; *p* < 0.001; C-statistic: 0.794 [0.731–0.856]). The addition of NLR, MLR, SII, or SIRI to the Global Registry of Acute Coronary Events (GRACE) risk score, especially SIRI (C-statistic: 0.699 [0.646–0.753], *p* < 0.001; net reclassification improvement [NRI]: 0.311 [0.209–0.407], *p* < 0.001; integrated discrimination improvement [IDI]: 0.024 [0.010–0.046], *p* < 0.001), outperformed the GRACE risk score alone in the risk predictive performance.

**Conclusion:**

Lymphocyte-based inflammatory indices were significantly and independently associated with MACE in ACS patients who underwent PCI. SIRI seemed to be better than the other four indices in predicting MACE, and the combination of SIRI with the GRACE risk score could predict MACE more accurately.

## Introduction

In previous studies, vulnerable plaques are generally considered to be the typical feature of acute coronary syndrome (ACS). Acute events caused by thrombosis after plaque rupture are considered to be the leading cause of death in patients with coronary artery disease (1). In recent years, plaque erosion has also been demonstrated to be one of the important causes of ACS (2–4). Compared with those with plaque rupture, patients with plaque erosion are more likely to develop non-ST segment elevation myocardial infarction (5). However, for patients with ST segment elevation myocardial infarction (STEMI), plaque rupture is still the major pathological factor in most patients (6).

The application of percutaneous coronary intervention (PCI) and the upgrading of interventional technologies and devices have significantly reduced the incidence of major adverse cardiovascular events (MACE), thereby improving the prognosis of patients with ACS (7). However, ACS patients undergoing PCI are still at high risk, and recurrent or persistent angina symptoms are still a thorny problem. By instantaneous wave-free ratio assessment, nearly one-quarter of patients still had residual ischemia after stent implantation (8), and 10.7% of patients were re-hospitalized within 30 days after procedure (9). Therefore, accurate and comprehensive risk assessment is particularly important in treatment decision-making for high-risk patients.

Inflammation plays an important role in the formation and development of atherosclerosis, and has been identified as a key harmful mediator and pathogenic factor of ischemia-reperfusion injury in STEMI patients (5, 10). Inflammatory cells like white blood cells and inflammation-related indices, such as platelet-lymphocyte ratio (PLR) and neutrophil-lymphocyte ratio (NLR), can affect the prognosis of ACS patients (11). These indices can be combined with the Global Registry of Acute Coronary Events (GRACE) risk score, the SYNergy between PCI with TAXus and cardiac surgery (SYNTAX) score, and other scores to improve the risk stratification ability for ACS patients (12). Two novel inflammatory markers, systemic immune inflammation index (SII) and system inflammation response index (SIRI), consisting of three blood routine markers, were first used to predict the prognosis of cancer (13, 14). Recently, their association with cardiovascular disease has attracted much attention. Studies have found that they can be used as risk stratification indices and predict adverse events (15). However, few studies have compared their predictive abilities with indices such as NLR. The lymphocyte-based inflammatory indices have attracted our attention because of their simple source and low cost. If they can predict the prognosis of ACS patients undergoing PCI, they will be good tools for stratifying patients at high risk.

The GRACE risk scoring system (16), which is widely used to predict the cumulative risk of death or myocardial infarction (MI) in ACS patients (17), includes age, heart rate, systolic blood pressure, creatinine, chronic heart failure, cardiac arrest at admission, ST-segment deviation, and elevated cardiac enzymes, but fails to involve any biological indicator. Therefore, we explored the ability of lymphocyte-based inflammatory indices in combination with the GRACE risk score to assess prognosis.

In this study, we evaluated the ability of five lymphocyte-based inflammatory indices including PLR, NLR, monocyte-lymphocyte ratio (MLR), SII, and SIRI to predict the long-term prognosis, and to improve the value of the GRACE risk score for risk stratification of ACS patients undergoing PCI.

## Method

### Study Design and Baseline Characteristics

This was a single-center prospective observational study based on cardiovascular center from Beijing Anzhen Hospital, Capital Medical University, which included 1,770 patients who underwent PCI for ACS between June 2016 and November 2017. We excluded 65 patients with at least one of the following conditions: prior coronary artery bypass grafting, acute and/or chronic infection, autoimmune diseases, known malignancy, Killip class > II, left ventricular ejection fraction <30%, or renal dysfunction with creatinine clearance <30 ml/min. Four patients were also excluded because of missing follow-up data despite at least four separate attempts to contact them. Finally, 1,701 patients were included in the analysis. All patients participating in the study were in line with the diagnostic criteria of ACS set by the American College of Cardiology Foundation/American Heart Association (ACC/AHA). This study was performed in accordance with the Helsinki Declaration of Human Rights and was approved by the institutional review board of Beijing Anzhen Hospital, Capital Medical University (IRB number: 2016034x).

### Measurements

Demographics, lifestyle, and clinical history were collected through standard questionnaires on admission. Body mass index was calculated based on height and weight [a ratio of weight to height squared (kg/m^2^)] of the patients on admission. The first peripheral venous blood after 12 h of fasting was obtained after admission at the hospital. Routine laboratory data and discharge medications were collected from the electronic medical system.

The counts of lymphocyte, platelet, neutrophil, and monocyte were measured in the Central Laboratory of Beijing Anzhen Hospital. In this study, the lymphocyte-based inflammatory indices included: PLR (platelet/ lymphocyte), NLR (neutrophil/ lymphocyte), MLR (monocyte/ lymphocyte), SII (platelet^*^ neutrophil/ lymphocyte), and SIRI (neutrophil^*^ monocyte/ lymphocyte) (15, 18). The GRACE risk score was analyzed as a numerical value and calculated according to the GRACE risk model by using a computer program (http://www.outcomes-umassmed.org/grace).

### Definition of Clinical Endpoints and Follow-Up

The primary endpoint of this study was the composite of all-cause death, non-fatal ischemic stroke, and non-fatal MI. Ischemic stroke was defined as ischemic cerebral infarction, clinically documented on brain computed tomography or magnetic resonance imaging. MI was defined as the appearance of new pathological Q waves in two or more contiguous leads, or the level of cardiac enzymes/markers exceeding the upper limit with either ischemic symptoms or electrocardiogram (ECG) implicating ischemia. However, within 1 week after the PCI, only new pathological Q-wave MI was defined as adverse event. The end of follow-up was the date of the first non-fatal MI or non-fatal ischemic stroke or all-cause death occurrence. If more than one event occurred, the most severe event was chosen (death > stroke > MI). Patients were followed up since the date of one month after discharge and every six months thereafter by telephone. Trained personnel who never knew the baseline data of patients achieved the telephone contact.

### Statistical Analysis

Statistical analyses were performed using the R, version 3.6.3 software (R Foundation for Statistical Computing, Vienna, Austria) and SPSS 24.0 (IBM Corporation, Chicago, IL). All statistical tests were two-tailed and *p* < 0.05 was considered statistically significant. Categorical variables were expressed as the percentage (number) tested with the chi-square test. Continuous variables were presented as mean with standard deviation or median with interquartile range (IQR). The normal distributions of the continuous variables were investigated by Kolmogorov-Smirnov test or histograms. Data with normal distribution were compared by ANOVA, otherwise by Kruskal-Wallis H tests. Receiver operating characteristic curves were used to calculate the cutoff values. The lymphocyte-based inflammatory indices were statistically analyzed as categorical variables according to the optimal cutoff values that were determined by Youden's index (sensitivity + specificity – 1). Univariate and multivariate Cox proportional hazards regression models were used to estimate the hazard ratio (HR) and 95% confidence interval (CI). The cumulative risk of the endpoint over time was presented graphically using Kaplan-Meier curve, and log-rank test was used to compare the two groups. To further evaluate the discrimination performance, the sensitivity, positive predictive value (PPV), and C-statistics were calculated, and C-statistics were compared pair-wise. Sensitivity refers to the probability of a positive laboratory test in a confirmed patient, and PPV refers to the probability of actual disease in a population with a positive laboratory test (19). To evaluate the ability of lymphocyte-based inflammatory indices to improve the predictive value of the GRACE risk model, we added these indices to the GRACE risk score as new models and performed net reclassification improvement (NRI) and integrated discrimination improvement (IDI) statistical analyses.

## Result

### Cohort Demographics

The mean age of the 1,701 patients at baseline was 60 ± 10 years, and 76.7% were men (*n* = 1,305). Among the 1,701 patients, more than one-half of the patients had hypertension (63.6%, *n* =1082), 46.0% (*n* = 783) had diabetes, 79.9% (*n* = 1,359) had dyslipidemia, and 12.8% (*n* = 218) were diagnosed as STEMI. During the median follow-up of 30 months (IQR, 30–36 months), 107 (6.3%) patients had MACE. Compared with those without event, patients with MACE had higher fasting plasma glucose levels, high-sensitivity C-reactive protein levels and SYNTAX score, but lower left ventricular ejection fraction. Also, patients with MACE had higher rate of aspirin and angiotensin-converting enzyme inhibitor (ACEI)/angiotensin II receptor blocker (ARB) use at discharge.

We sorted out the relevant variables of the GRACE risk model. Except creatinine and cardiac arrest, other GRACE variables were significantly different between patients with and without MACE. Compared with those without MACE, patients with MACE had higher GRACE risk scores, and had higher levels of NLR, PLR, MLR, SII, and SIRI. Baseline characteristics of the study population are summarized in Table 1.

**Table 1:** Baseline characteristics of study population by major adverse cardiovascular events (MACE).

**Variable**	**Total study population**	**No such event**	**MACE**	***P*** **value**
	***N*** **= 1,701**	***N*** **= 1,594**	***N*** **= 107**	
Male, *n* (%)	1,305 (76.7)	1,227 (77.0)	78 (72.9)	0.334
BMI (kg/m^2^)	25.7 ± 9.6	25.7 ± 9.4	25.3 ± 11.4	0.218
Current smoking, *n* (%)	754 (44.3)	709 (44.5)	45 (42.1)	0.625
Hypertension, *n* (%)	1082 (63.6)	1107 (63.2)	75 (70.1)	0.150
Diabetes, *n* (%)	783 (46.0)	731 (45.9)	52 (48.6)	0.582
Dyslipidemia, *n* (%)	1359 (79.9)	1268 (79.5)	91 (85.0)	0.170
Previous MI, *n* (%)	325 (19.1)	294 (18.4)	31 (29.0)	0.007
Previous PCI, *n* (%)	338 (19.9)	304 (19.1)	34 (31.8)	0.001
CKD, *n* (%)	737 (43.3)	684 (42.9)	53 (49.5)	0.181
Type of ACS
UA, *n* (%)	1267 (74.5)	1196 (75.0)	71 (66.4)	0.046
NSTEMI, *n* (%)	216 (12.7)	193 (12.1)	23 (21.5)	0.005
STEMI, *n* (%)	218 (12.8)	205 (12.9)	13 (12.1)	0.831
GRACE variables
Age (years)	60 ± 10	59 ± 10	64 ± 12	<0.001
HR (bpm)	68 ± 9	68 ± 9	73 ± 10	<0.001
SBP (mmHg)	130 ± 16	130 ± 16	134 ± 18	0.006
Creatinine (μmol/L)	70.3 [62.1–79.6]	70.3 [62.0–79.4]	70.2 [63.2–81.3]	0.432
Heart failure, *n* (%)	115 (6.8)	89 (5.6)	26 (24.3)	<0.001
ST-segment deviation, *n* (%)	298 (17.5)	269 (16.9)	29 (27.1)	0.007
Elevated cardiac enzymes/markers, *n* (%)	434 (25.5)	398 (25.0)	36 (33.6)	0.046
Cardiac arrest, *n* (%)	2 (0.1)	2 (0.1)	0 (0.0)	1.000
GRACE risk score	103 ± 38	102 ± 37	121 ± 44	<0.001
Laboratory data
Triglycerides (mmol/L)	1.45 [1.01–2.06]	1.44 [1.00–2.04]	1.54 [1.12–2.28]	0.120
Total cholesterol (mmol/L)	4.15 ± 0.99	4.14 ± 0.99	4.24 ± 1.02	0.309
HDL-C (mmol/L)	1.03 ± 0.23	1.04 ± 0.23	1.00 ± 0.25	0.128
LDL-C (mmol/L)	2.44 ± 0.81	2.44 ± 0.81	2.51 ± 0.80	0.337
FPG (mmol/L)	5.78 [5.23–6.92]	5.78 [5.22–6.87]	6.12 [5.33–7.45]	0.018
hsCRP (mg/L)	1.34 [0.64–3.42]	1.32 [0.62–3.23]	2.86 [1.16–7.14]	<0.001
LVEF (%)	65 [60–68]	65 [60–68]	60 [53–66]	<0.001
30–39, *n* (%)	21 (1.2)	16 (1.0)	5 (4.7)	
40–49, *n* (%)	74 (4.4)	61 (3.8)	13 (12.1)	
≥50, *n* (%)	1606 (94.4)	1517 (95.2)	89 (83.2)	
Angiographic and procedural results
Left-main/multi-vessel disease, *n* (%)	1,441 (84.7)	1347 (84.5)	94 (87.9)	0.352
Proximal LAD disease, *n* (%)	850 (50.0)	790 (49.6)	60 (56.1)	0.192
SYNTAX score	20 ± 11	20 ± 11	24 ± 12	<0.001
DES, *n* (%)	1397 (82.1)	1307 (82.0)	90 (84.1)	0.580
BRS, *n* (%)	97 (5.7)	91 (5.7)	6 (5.6)	0.965
DCB, *n* (%)	82 (4.8)	76 (4.8)	6 (5.6)	0.923
Discharge medications
Aspirin, *n* (%)	1685 (99.1)	1586 (99.5)	99 (92.5)	<0.001
P2Y12 inhibitors, *n* (%)	1701 (100)	1594 (100)	107 (100)	/
Statins, *n* (%)	1701 (100)	1594 (100)	107 (100)	/
ACEI/ARBs, *n* (%)	821 (48.3)	748 (46.9)	73 (68.2)	<0.001
β-blockers, *n* (%)	1197 (70.4)	1123 (70.5)	74 (69.2)	0.777
Lymphocyte-based inflammatory indices
PLR	118.06 [94.15–150.00]	117.20 [93.61–148.66]	137.74 [100.00–172.31]	0.002
NLR	2.26 [1.72–2.97]	2.22 [1.70–2.92]	2.91 [2.05–3.80]	<0.001
MLR	0.20 [0.16–0.26]	0.20 [0.15–0.26]	0.25 [0.19–0.32]	<0.001
SII	468.00 [339.94–644.45]	461.51 [336.50–630.71]	613.42 [423.45–938.94]	<0.001
SIRI	0.80 [0.55–1.17]	0.78 [0.55–1.12]	1.20 [0.81–1.77]	<0.001

*ACEI, angiotensin converting enzyme inhibitor; ACS, acute coronary syndrome; ARB, angiotensin II receptor blocker; BMI, body mass index; BRS, bioresorbable scaffold; CKD, chronic kidney disease; DCB, drug coated balloon; DES, drug eluting stent; FPG, fasting plasma glucose; GRACE, global registry of acute coronary events; HDL-C, high-density lipoprotein-cholesterol; HR, heart rate; HsCRP, high-sensitive C-reaction protein; LAD, left anterior descending branch; LDL-C, low-density lipoprotein-cholesterol; LVEF, left ventricular ejection fraction; MLR, monocyte-lymphocyte ratio; MI, myocardial infarction; NLR, neutrophil-lymphocyte ratio; NSTEMI, non-ST segment elevation myocardial infarction; PCI, percutaneous coronary intervention; PLR, platelet-lymphocyte ratio; SBP, systolic blood pressure; STEMI, ST segment elevation myocardial infarction; SII, systemic inflammatory reaction index; SIRI, systemic inflammatory response index; SYNTAX, Synergy between PCI with TAXus and cardiac surgery; UA, unstable angina*.

### Lymphocyte-Based Inflammatory Indices as Independent Predictors of MACE

**Figure 1:**
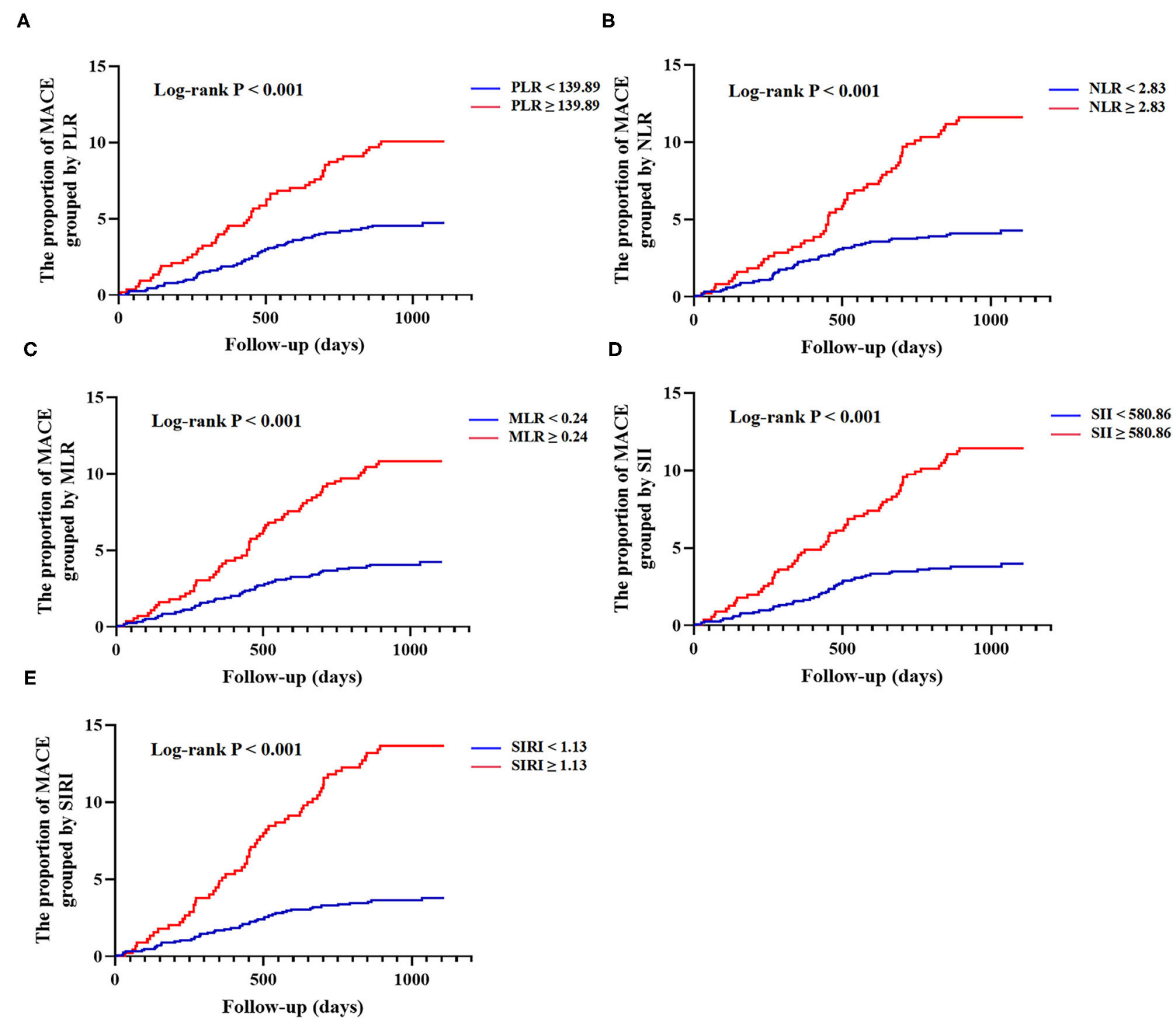
Kaplan-Meier curves of lymphocyte-based inflammatory indices and cumulative incidence of major adverse cardiovascular events (MACE) at follow-up. **(A)** Grouped by platelet-lymphocyte ratio (PLR) (<139.89 vs. ≥139.89); **(B)** Grouped by neutrophil-lymphocyte ratio (NLR) (<2.83 vs. ≥2.83); **(C)** Grouped by monocyte-lymphocyte ratio (MLR) (<0.24 vs. ≥0.24); **(D)** Grouped by systemic immune inflammation index (SII) (<580.86 vs. ≥580.86); **(E)** Grouped by system inflammation response index (SIRI) (<1.13 vs. ≥1.13). MACE was defined as a composite of all-cause death, non-fatal ischemic stroke, and non-fatal myocardial infarction.

The results of univariate and multivariate Cox proportional hazards regression analyses of lymphocyte-based inflammatory indices predicting MACE are summarized in Table 2. The univariate COX analysis showed higher rates of MACE corresponding to higher PLR (HR: 2.234; 95% CI: 1.530–3.264; *p* < 0.001), NLR (HR: 2.852; 95% CI: 1.951–4.169; *p* < 0.001), MLR (HR: 2.641; 95% CI: 1.794–3.887; *p* < 0.001), SII (HR: 3.055; 95% CI: 2.079–4.490; *p* < 0.001), and SIRI (HR: 3.847; 95% CI: 2.623–5.641; *p* < 0.001). In the multivariate COX analysis, the associations of PLR (HR: 1.768; 95% CI: 1.186–2.636; *p* = 0.005), NLR (HR: 1.767; 95% CI: 1.163–2.685; *p* = 0.008), MLR (HR: 1.795; 95% CI: 1.185–2.719; *p* = 0.006), SII (HR: 2.241; 95% CI: 1.471–3.414; *p* < 0.001), and SIRI (HR: 2.561; 95% CI: 1.681–3.902; *p* < 0.001) with MACE remained significant. As shown in Figure 1, Kaplan-Meier curves showed that the patients with higher lymphocyte-based inflammatory indices had higher incidences of MACE (all log-rank *p* < 0.001) (Supplementary Table 1).

**Table 2:** The univariate and multivariate Cox proportional hazards analyses of lymphocyte-based inflammatory indices predicting MACE.

	**Univariate**		Multivariate*	
	**HR (95% Cl)**	***P*** **Value**	**HR (95% Cl)**	***P*** **Value**
PLR	2.234 (1.530–3.264)	<0.001	1.768 (1.186–2.636)	0.005
NLR	2.852 (1.951–4.169)	<0.001	1.767 (1.163–2.685)	0.008
MLR	2.641 (1.794–3.887)	<0.001	1.795 (1.185–2.719)	0.006
SII	3.055 (2.079–4.490)	<0.001	2.241 (1.471–3.414)	<0.001
SIRI	3.847 (2.623–5.641)	<0.001	2.561 (1.681–3.902)	<0.001

* *Adjusted for GRACE risk score, past MI, past PCI, type of ACS, FPG, hsCRP, LVEF, SYNTAX score, use of aspirin and ACEI/ARBs at discharge*.

*Abbreviations as in Table 1*.

### Comparisons Among Various Lymphocyte-Based Inflammatory Indices

The comparisons among various lymphocyte-based inflammatory indices for predicting MACE are shown in Table 3. We observed that the sensitivity of MLR was the highest (59.1%), and the PPV of SIRI was the highest (13.4%). The C-statistics of the lymphocyte-based inflammatory indices were 0.692 [0.611–0.773] for PLR, 0.739 [0.666–0.812] for NLR, 0.729 [0.654–0.805] for MLR, 0.754 [0.682–0.825] for SII, and 0.794 [0.731–0.856] for SIRI. According to pair-wise comparison of the C-statistics, SIRI seemingly performed best.

**Table 3:** Comparisons among various lymphocyte-based inflammatory indices.

**A**.					
**Discrimination ability**	**PLR**	**NLR**	**MLR**	**SII**	**SIRI**
Cutoff value	139.89	2.83	0.24	580.86	1.13
Sensitivity (%)	48.9	52.8	59.1	58.2	56.4
Positive predictive value (%)	9.9	11.4	10.9	11.3	13.4
C-statistic (95% CI)	0.692 [0.611–0.773]	0.739 [0.666–0.812]	0.729 [0.654–0.805]	0.754 [0.682–0.825]	0.794 [0.731–0.856]
**B**.		
	**C-statistic**	
**Comparison**	**Difference**	***P*** **Value**
NLR VS. PLR	0.047	0.128
NLR VS. MLR	0.010	0.402
MLR VS. PLR	0.037	0.218
SII VS. PLR	0.061	0.042
SII VS. NLR	0.014	0.317
SII VS. MLR	0.024	0.283
SIRI VS. PLR	0.101	0.013
SIRI VS. NLR	0.054	0.044
SIRI VS. MLR	0.064	0.018
SIRI VS. SII	0.040	0.114

*(A) The discrimination ability of five lymphocyte-based inflammatory indices; (B) The pair-wise comparison of C-statistics among five lymphocyte-based inflammatory indices*.

*Abbreviations as in Table 1*.

### Combinations of Lymphocyte-Based Inflammatory Indices With the GRACE Risk Score

To assess whether the combinations of lymphocyte-based inflammatory indices with the GRACE risk score could improve the predictive ability, we built six models with the GRACE risk score numerically incorporated into the models (Table 4). Compared with the basic model, the risk models consisting of the GRACE risk score and lymphocyte-based inflammatory indices had superior discrimination performance for MACE. We observed that the C-statistics increased significantly after adding NLR (0.668 [0.612–0.724], *p* = 0.018), MLR (0.672 [0.619–0.725], *p* = 0.010), SII (0.680 [0.627–0.733], *p* = 0.005), and SIRI (0.699 [0.646–0.753], *p* < 0.001) to the GRACE risk score. Among the five new models, the model with the GRACE risk score in combination with SIRI had the best reclassification significance with NRI of 31.1% (*p* < 0.001) and IDI of 2.4% (*p* < 0.001).

**Table 4:** Discrimination performance of GRACE risk score plus lymphocyte-based inflammatory indices in predicting MACE.

**Model**	**C-Statistic (95% Cl)**	***P*** **value**	**NRI**	***P*** **value**	**IDI**	***P*** **value**
GRACE	0.624 [0.566–0.682]	ref	ref		ref	
GRACE + PLR	0.656 [0.602–0.710]	0.057	0.199 [0.104–0.297]	<0.001	0.009 [0.002–0.024]	<0.001
GRACE + NLR	0.668 [0.612–0.724]	0.018	0.250 [0.148–0.341]	<0.001	0.015 [0.004–0.030]	<0.001
GRACE + MLR	0.672 [0.619–0.725]	0.010	0.245 [0.143–0.342]	0.002	0.011 [0.002–0.027]	0.002
GRACE + SII	0.680 [0.627–0.733]	0.005	0.268 [0.162–0.361]	<0.001	0.015 [0.005–0.031]	<0.001
GRACE + SIRI	0.699 [0.646–0.753]	<0.001	0.311 [0.209–0.407]	<0.001	0.024 [0.010–0.046]	<0.001

*Abbreviations as in Table 1*.

## Discussion

In this observational study, we evaluated the prognostic values of five lymphocyte-based inflammatory indices in ACS patients who underwent PCI for the first time. Lymphocyte-based inflammatory indices are readily available in clinical practice. We observed that the five indices were significantly and independently associated with MACE in ACS patients. Through univariate and multivariate analysis, SIRI showed the highest C-statistics (0.794; 0.699), affirming the predictive value of SIRI. Although the C-statistic of SIRI was higher in univariate analysis, it did not mean that the predictive value of SIRI alone was higher. In multivariate analysis, multiple influencing or confounding factors were comprehensively considered.

In previous studies, NLR attracted the most attention from researchers. A number of studies showed that NLR promoted the development of atherosclerosis. Choi et al. found that NLR > 2.8 was an independent predictor of adverse cardiovascular events in patients with CAD undergoing PCI (20). Our study had a similar result that NLR ≥ 2.83 could predict the occurrence of MACE. The study of XU et al. showed a significant increase in 2-year adverse cardiovascular events in patients with left main and/or three-vessel disease when NLR ≥ 3.39 (21). An increase in neutrophils can promote oxidative damage to the vessel wall, while a decrease in lymphocytes can also exacerbate oxidative and inflammatory damage, both of which are associated with increased stiffness of the arteries (22, 23). NLR has been shown to be independently associated with coronary artery calcification, which increases the risk of CAD (24). Of note, even after receiving dual antiplatelet therapy, ACS patients with high NLR levels still have poor platelet inhibition, which promotes thrombosis and increases the risk of recurrent ischemic events (25).

Elevated PLR levels may be related to inflammatory activation and pro-thrombotic status in patients with ACS due to megakaryocyte proliferation and relative prothrombotic status (26). Li and colleagues observed that PLR significantly increased in elderly patients, resulting in poor prognosis (27). The study of Trakarnwijitr et al. showed that PLR was an independent risk factor for CAD in patients aged 55 years and above, but was negatively associated with CAD in younger patients (28). The mean age of patients in our study was 60 ± 10 years, and we found that PLR has a limited prognostic value in ACS patients. Based on the results of our study, we do not recommend using PLR alone to predict cardiovascular outcomes, but we may consider combining PLR with other indices for risk stratification. The study of Liu et al. indicated that PLR-NLR combination could better predict the prognosis of acute MI and had higher sensitivity than PLR or NLR alone (29).

One study showed that MLR was independently associated with CAD and could be used to predict coronary lesion severity (30). The study of Song et al. yielded similar results (31). As one of the most important inflammatory cells, monocytes are directly involved in the formation and development of atherosclerosis. Monocytes adhere to vascular endothelium and differentiate into macrophages, and then transform into foam cells by ingesting oxidized lipoprotein, which can activate various inflammatory signal factors and oxidized free radicals in plaque (32, 33). It is encouraging that therapies targeting monocytes, macrophages, and foam cells are available to treat atherosclerosis (34).

SII and SIRI were originally used to evaluate the prognosis of tumors. In recent years, SII and SIRI have been shown to be good predictors of CAD. In fact, SII and SIRI are more comprehensive because both are a combination of three inflammatory cells compared to the other three indices. Therefore, it is not surprising that SII and SIRI outperformed the other three indices in predicting cardiovascular outcomes. Of note, in addition to neutrophil and lymphocyte, the other component included in SII is platelet, while in SIRI it is monocyte. Monocyte may be more closely related to the development of atherosclerosis than platelet. SII was shown to be positively correlated with SYNTAX score (35, 36), which could be used for CAD risk stratification and prognostic prediction after PCI. Jin and colleagues found that the high rates of stroke and all-cause death corresponded to high levels of SII and SIRI, while the high risk of MI was only independently related to high SIRI (15). In the present study, we demonstrated that the predictive ability of SIRI for MACE was better than SII.

The GRACE scoring system is relatively common, standardized, and authoritative. The GRACE risk score combined with other indicators (such as platelet reactivity, hemoglobin A1c, and red blood cell distribution width) had a better predictive value than the GRACE risk score alone (37–39). One of our purposes is to explore the ability of five indicators to improve the GRACE score. Previous studies showed that adding neutrophil count to the GRACE risk score increased the C-statistic (0.698 vs. 0.796, *p* < 0.001), and enhanced the NRI (0.637, *p* = 0.020) and IDI (0.180, *p* < 0.001) (40). Similar results were obtained by Zhou et al., where the GRACE risk score combined with NLR improved the C- statistic (0.69 vs. 0.77) (41). In our study, adding NLR to the GRACE risk score also increased the C-statistic (0.624 vs. 0.668), as well as enhanced the levels of NRI (0.250, *p* < 0.001) and IDI (0.015, *p* < 0.001). However, few studies investigated whether the addition of the other four indices, particularly SII and SIRI (higher predictive value in univariate analysis), improves the predictive ability of the GRACE risk score. In our study, for the first time, we added five lymphocyte-based inflammatory indices to the GRACE risk score, further illustrating their respective predictive ability, and we found that the addition of NLR, MLR, SII, or SIRI to the GRACE risk score, especially SIRI, outperformed the GRACE risk score alone in the risk predictive performance.

## Limitation

The present study has some limitations. First, this was a single-center study with a relatively small sample size. Second, our study was limited to Chinese subjects, and thus the conclusion requires further validation before extending to other ethnic groups. Third, our study did not investigate the correlation between the lymphocyte-based inflammatory indices with the severity of CAD in ACS patients, which needs to be explored in subsequent studies.

## Conclusion

The values of PLR ≥ 139.89, NLR ≥ 2.83, MLR ≥ 0.24, SII ≥ 580.86, and SIRI ≥ 1.13 were significantly and independently associated with MACE in ACS patients who underwent PCI. SIRI seemed to be better than the other four indices in predicting MACE, and the combination of SIRI with the GRACE risk score could predict MACE more accurately. In the future, we can add the SIRI as a categorical variable to the GRACE risk score to complement the inflammation deficit. As for the classification threshold or the score weight in the GRACE scoring system of SIRI, further exploration is required.

## Data Availability Statement

The raw data supporting the conclusions of this article will be made available by the authors, without undue reservation.

## Ethics Statement

The studies involving human participants were reviewed and approved by the Institutional Review Board of Beijing Anzhen Hospital, Capital Medical University (IRB number: 2016034x). The patients/participants provided their written informed consent to participate in this study.

## Author Contributions

All authors were involved in the conception and design of the study, the collection, analysis, interpretation of the data, reviewed the final manuscript, read, and approved the final manuscript.

## Funding

This work was supported by China Postdoctoral Science Foundation (2021M692253), Beijing Postdoctoral Research Foundation (2021-ZZ-023), and Beijing Municipal Administration of Hospitals' Mission Plan (SML20180601).

## Conflict of Interest

The authors declare that the research was conducted in the absence of any commercial or financial relationships that could be construed as a potential conflict of interest.

## Publisher's Note

All claims expressed in this article are solely those of the authors and do not necessarily represent those of their affiliated organizations, or those of the publisher, the editors and the reviewers. Any product that may be evaluated in this article, or claim that may be made by its manufacturer, is not guaranteed or endorsed by the publisher.

## Abbreviations

ACS: acute coronary syndrome
CI: confidence interval
GRACE: Global Registry of Acute Coronary Events
IDI: integrated discrimination improvement
IQR: interquartile range
MACE: major adverse cardiovascular events
MLR: monocyte-lymphocyte ratio
MI: myocardial infarction
NRI: net reclassification improvement
NLR: neutrophil-lymphocyte ratio
PCI: percutaneous coronary intervention
PLR: platelet-lymphocyte ratio
PPV: positive predictive value
SYNTAX: SYNergy between PCI with TAXus and cardiac surgery
SII: systemic inflammatory reaction index
SIRI: systemic inflammatory response index.
