# Supplementary material for: Prognostic Impact of Multiple Lymphocyte-Based Inflammatory Indices in Acute Coronary Syndrome Patients
Source: Front Cardiovasc Med. 2022 May 3;9:811790. doi: 10.3389/fcvm.2022.811790 (PMC9110784; doi:10.3389/fcvm.2022.811790)
Supplement: Supplementary file 1 [file Table_1.pdf]

Supplement Table. The occurrence of each clinical outcomes in different groups of lymphocyte-based inflammatory indices

MACE (N=107)		Clinical outcomes				
		**All-cause death (n=43)**	**Cardiovascular death (n=36)**	**Non-cardiovascular death (n=7)**	**Non-fatal MI (n=49)**	**Non-fatal ischemic stroke (n=24)**
**PLR**	**<139.89**	13 (30.2%)	12 (33.3%)	1 (14.3%)	28 (57.1%)	15 (62.5%)
	**≥139.89**	30 69.8%)	24 (66.7%)	6 (85.7%)	21 (42.9%)	9 (37.5%)
**NLR**	**<2.83**	10 (23.3%)	9 (25.0%)	1 (14.3%)	27 (55.1%)	13 (54.2%)
	**≥2.83**	33 (76.7%)	27 (75.0%)	6 (85.7%)	22 (44.9%)	11 (45.8%)
**MLR**	**<0.24**	11 (25.6%)	10 (27.8%)	1 (14.3%)	25 (51.0%)	13 (54.2%)
	**≥0.24**	32 (74.4%)	26 (72.2%)	6 (85.7%)	24 (49.0%)	11 (45.8%)
**SII**	**<580.86**	9 (20.9%)	8 (22.2%)	1 (14.3%)	23 (46.9%)	12 (50.0%)
	**≥580.86**	34 (79.1%)	28 (77.8%)	6 (85.7%)	26 (53.1%)	12 (50.0%)
**SIRI**	**<1.13**	12 (27.9%)	11 (30.6%)	1 (14.3%)	24 (49.0%)	11 (45.8%)
	**≥1.13**	31 (72.1%)	25 (69.4%)	6 (85.7%)	25 (51.0%)	13 (54.2%)

Abbreviations as in Table 1.
